# High MAST2 mRNA expression and its role in diagnosis and prognosis of liver cancer

**DOI:** 10.1038/s41598-019-56476-x

**Published:** 2019-12-27

**Authors:** Yan Jiao, Yanqing Li, Peiqiang Jiang, Zhuo Fu, Yahui Liu

**Affiliations:** 1 Department of Hepatobiliary and Pancreatic Surgery, The First Hospital of Jilin University, Changchun, Jilin, 130021 P.R. China; 2 Department of Pathophysiology, College of Basic Medical Sciences, Jilin University, Changchun, Jilin, 130021 P.R. China; 3 Department of Hand and Foot Surgery, The First Hospital of Jilin University, Changchun, Jilin, 130021 P.R. China

**Keywords:** Diagnostic markers, Prognostic markers

## Abstract

Liver cancer is a high morbidity and low survival disease all over the world. Chromosomal instability is hallmark of liver cancer. Microtubule-associated serine and threonine kinase 2 (MAST2), as a microtubule associated protein, may involve in tumorous chromosomal instability and plays important roles in cell proliferation and survival. The role of MAST2 in liver cancer has not been well elucidated, which is the aim of our study. In this study, The Cancer Genome Atlas database was used to study the MAST2 mRNA expression in liver cancer, and Chi-squared tests were performed to test the correlation between clinical features and MAST2 expression. ROC curve was performed to examined the diagnostic capacity. The prognostic value of MAST2 in liver cancer was assessed through Kaplan–Meier curves as well as Cox analysis. Our results showed MAST2 was upregulated in liver cancer, and the area under the curve (AUC) was 0.925 and indicated powerful diagnostic capability. High MAST2 expression was associated with advanced clinical status such as histological type (*p* = 0.0059), histologic grade (*p* = 0.0142), stage (*p* = 0.0008), T classification (*p* = 0.0028), N classification (*p* = 0.0107), survival status (*p* = 0.0062), and poor prognosis of patients. Importantly, MAST2 was an independent risk factor for patients’ prognosis after adjusting for other risk factors including stage, T classification, and residual tumor. In total, MAST2 is a potential diagnostic and prognostic biomarker of liver cancer.

## Introduction

Cancer is a major problem in public health in the world. Liver cancer, a highly fatal cancer, is estimated to account for about 42030 new cancer cases and 31780 cancer deaths in the United States in 2019^1^. Liver cancer is one of the lowest survival cancers, which is predominantly due to the fact that diagnosis is often made late or inaccurate^2^. Therefore, to identify a new biomarker for ea--rly and accurate diagnosis has great clinical significance.

Chromosomal instability is a hallmark for carcinoma. As a novel gene family which may involve in chromosomal instability, MAST functions in normal cell division. Its alterations lead to a few mitotic abnormalities, such as spindle malformation, chromosome missegregation, centrosome amplification, and failure of cytokinesis^3^. Furthermore, overexpression of MAST2 gene has a proliferative effect both *in vitro* and *in vivo*^4^. Microtubule-associated serine and threonine kinase 2 (MAST2) is a 205 kD protein that is associated with microtubules^5^. MAST2 interacts with the carboxyl-terminal of phosphatase and tensin homolog (PTEN) through its PDZ (PSD-95, Dlg1, Zo-1) domain^6^. They are crucial for cell division, survival and tumorigenesis^7^. However, until now, little is known about MAST gene family. The specific role of MAST2 in liver cancer needs more elucidation.

In this study, we compared MAST2 expression in liver cancer patients and then evaluated its diagnostic value. We also analyzed the relationship between clinical variables of patients and MAST2 expression, and further explored the prognostic value of MAST2 in patients’ overall survival (OS) and relapse-free survival (RFS). Our study demonstrated that MAST2 could become a novel diagnostic and prognostic biomarker for liver cancer patients.

## Results

### High MAST2 expression in liver cancer

**Figure 1:**
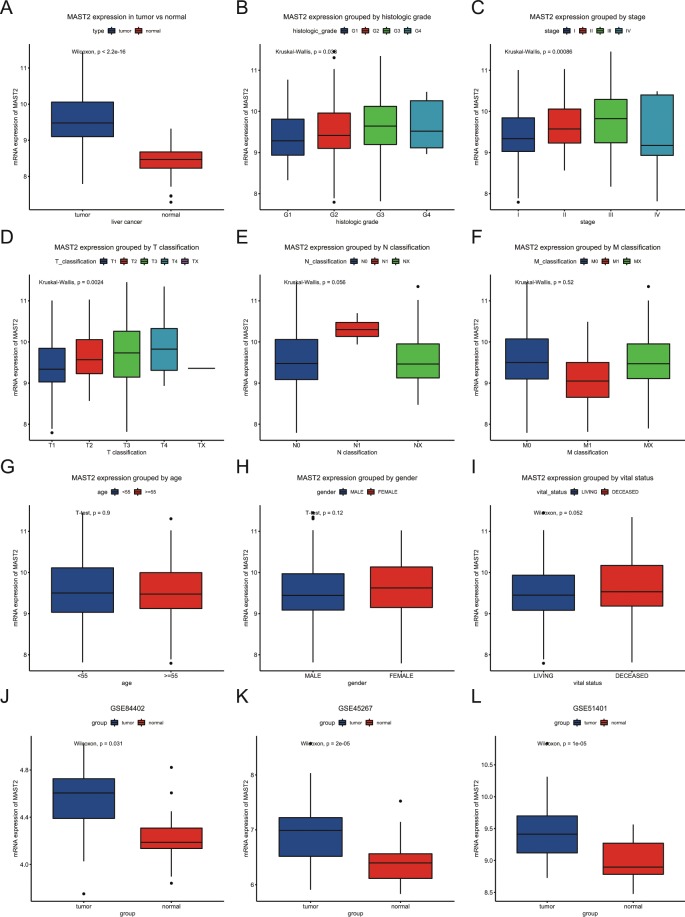
MAST2 expression in liver cancer. MAST2 expression was compared between normal tissues and liver cancer tissues. Subgroup analysis for histologic grade, stage, T classification, N classification, M classification, age, gender and vital status. The expression of MAST2 was verified by GEO datasets including GSE84402, GSE45267, GSE51401.

A total of 373 liver cancer patients were included. The detailed characteristics, including age, gender, stage, classifications, were shown in Table 1. Boxplots showed the differences in MAST2 expression by tumor vs adjacent normal tissue (Fig. 1A). The results in Fig. 1A demonstrated MAST2 expression was higher in tumors (*p* < 22e-16), which were also verified by GEO datasets including GSE84402, GSE45267, GSE51401 (Fig. 1J–L). Moreover, the expression of MAST2 was also distinct in subgroups of histologic grade (*p* = 0.03), stage (*p* = 0.00086), T classification (p = 0.0024). Higher histological grades (except G4), higher stages (except stage IV) and T classification have higher MAST2 expression. However, there were no significant differences in MAST2 expression between subgroups divided by N classification, M classification, age, gender and vital status (Fig. 1D–I).

**Table 1:** Clinical characteristics.

characteristics	Number	%
**age**		
<55	117	31.45
>=55	255	68.55
not appplicable	1	0.00
**gender**		
FEMALE	121	32.44
MALE	252	67.56
Histological_type		
Fibrolamellar_Carcinoma	3	0.8
Hepatocellular_Carcinoma	363	97.32
Hepatocholangiocarcinoma	7	1.88
**Histologic_grade**		
Grade_1	55	14.75
Grade_2	178	47.72
Grade_3	123	32.98
Grade_4	12	3.22
not appplicable	5	1.34
**clinical_stage**		
stage_I	172	46.11
stage_II	87	23.32
stage_III	85	22.79
stage_IV	5	1.34
not appplicable	24	6.43
**T_classification**		
T1	182	48.79
T2	95	25.47
T3	80	21.45
T4	13	3.49
Tx	1	0.27
not appplicable	2	0.54
**N_classification**		
N0	253	67.83
N1	4	1.07
Nx	115	30.83
not appplicable	1	0.27
**M_classification**		
M0	267	71.58
M1	4	1.07
Mx	102	27.35
**Radiation_therapy**		
NO	340	91.15
YES	8	2.14
not appplicable	25	6.7
**Residual_tumor**		
R0	326	87.4
R1	17	4.56
R2	1	0.27
Rx	22	5.9
not appplicable	7	1.88
**survival_status**		
DECEASED	130	34.85
LIVING	243	65.15
**relapse**		
NO	179	55.94
YES	141	44.06
**MAST2**		
high	110	29.49
low	263	70.51

Note: The table is partly similarity with previous publications in form^8–11^.

### The diagnostic potential of MAST2

**Figure 2:**
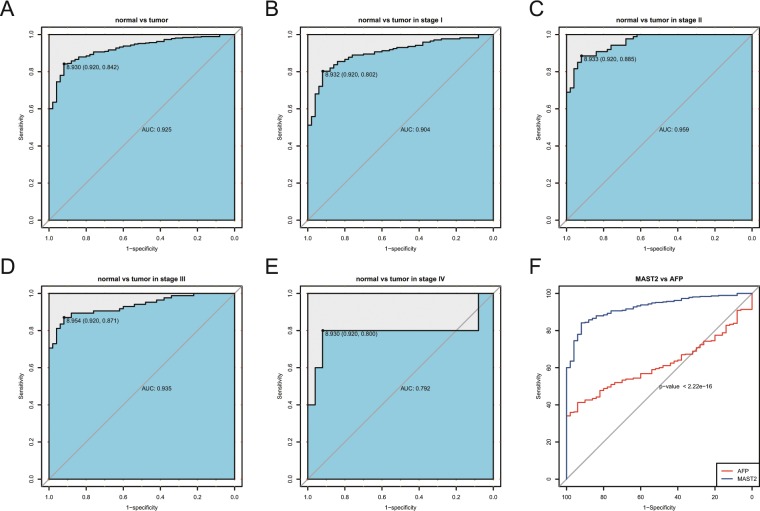
Diagnostic value of MAST2 expression in liver cancer. ROC for expression of MAST2 in normal tissues and liver cancer. Subgroup analysis for stage I, II, III and IV. ROC for MAST2 vs AFP.

ROC showed the diagnostic capability of MAST2 (Fig. 2). The area under the curve (AUC) was 0.925 between tumor and normal tissues, which represented a powerful diagnostic capability (Fig. 2A). We further performed ROC analysis in subgroup of different stage, which also showed moderate to high diagnostic capability (stage I: 0.904; stage α: 0.959; stage III: 0.935; stage IV: 0.792; Fig. 2B–E). In addition, we compared the diagnostic value of MAST2 and AFP through ROC curve and found MAST2 had more diagnostic value (Fig. 2F).

### The relationship between characteristics of patients and MAST2 expression

Table 2 summarized the association between clinical variables and MAST2 expression. Results showed MAST2 expression was significantly associated with histological type (*p* = 0.0059), histologic grade (*p* = 0.0142), stage (*p* = 0.0008), T classification (*p* = 0.0028), N classification (*p* = 0.0107), and survival status (*p* = 0.0062).

**Table 2:** Relationship between clinical variables and MAST2 expression.

Characteristics	Variable	Number	MAST2 expression				χ2	p-value
			High	%	Low	%		
age	<55	117	38	34.55	79	30.15	0.5046	0.4775
	>=55	255	72	65.45	183	69.85		
gender	FEMALE	121	42	38.18	79	30.04	1.9902	0.1583
	MALE	252	68	61.82	184	69.96		
histological_type	Fibrolamellar Carcinoma	3	3	2.73	0	0	9.9642	**0.0069**
	Hepatocellular Carcinoma	363	103	93.64	260	98.86		
	Hepatocholangiocarcinoma (Mixed)	7	4	3.64	3	1.14		
histologic_grade	Grade_1	55	9	8.18	46	17.83	10.1341	**0.0142**
	Grade_2	178	49	44.55	129	50		
	Grade_3	123	47	42.73	76	29.46		
	Grade_4	12	5	4.55	7	2.71		
clincial_stage	stage_I	172	36	34.62	136	55.51	15.9814	**0.0008**
	stage_II	87	28	26.92	59	24.08		
	stage_III	85	38	36.54	47	19.18		
	stage_IV	5	2	1.92	3	1.22		
T_classification	T1	182	39	35.45	143	54.79	14.7546	**0.0028**
	T2	95	31	28.18	64	24.52		
	T3	80	34	30.91	46	17.62		
	T4	13	6	5.45	7	2.68		
	Tx	1	0	0	1	0.38		
N_classification	N0	253	75	68.81	178	67.68	10.2393	**0.0107**
	N1	4	4	3.67	0	0		
	Nx	115	30	27.52	85	32.32		
M_classification	M0	267	82	74.55	185	70.34	0.6776	0.7702
	M1	4	1	0.91	3	1.14		
	Mx	102	27	24.55	75	28.52		
radiation_therapy	NO	340	100	98.04	240	97.56	0	1
	YES	8	2	1.96	6	2.44		
residual_tumor	R0	326	94	86.24	232	90.27	3.1493	0.3858
	R1	17	5	4.59	12	4.67		
	R2	1	0	0	1	0.39		
	Rx	22	10	9.17	12	4.67		
survival_status	DECEASED	130	50	45.45	80	30.42	7.075	**0.0078**
	LIVING	243	60	54.55	183	69.58		

Note: Bold values represent *p* < 0.05. The table is partly similarity with previous publications in form^8–11^.

### MAST2 expression is associated with OS

**Figure 3:**
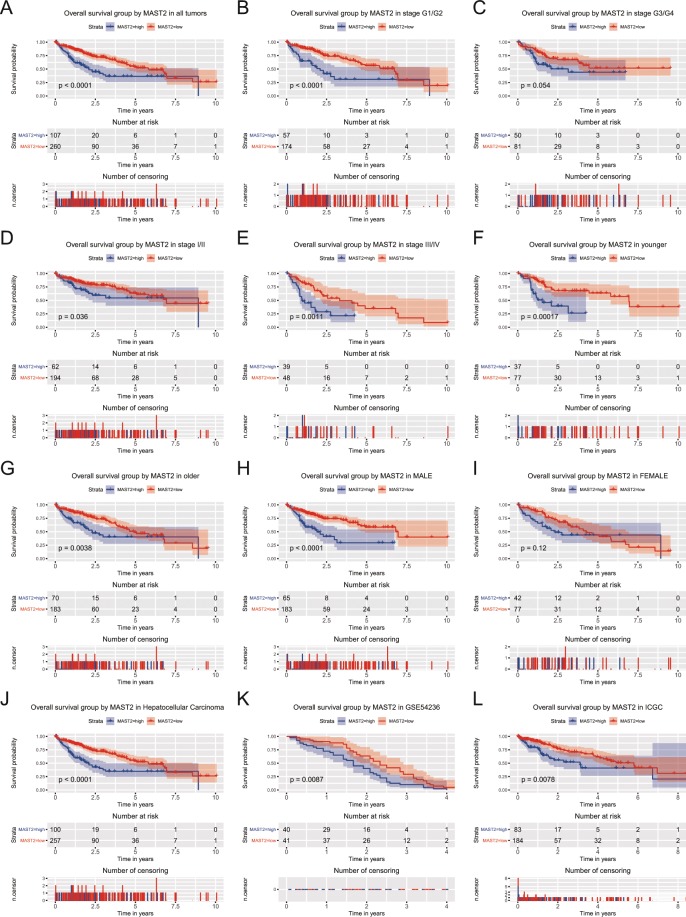
Kaplan-Meier curves for OS in liver cancer. Kaplan-Meier curves for OS in liver cancer for all patients, and patients in subgroup of stage G1/G2, stage G3/G4, stage I/II, stage III/IV, younger, older, male, female and HCC. The verification in GSE54236 and ICGC.

Proper threshold from ROC curve was cutoff to divided patients into two groups (high and low MAST2 expression). Kaplan-Meier curves were used to estimate the prognostic role of MAST2 in patients with liver cancer (Fig. 3). Results showed patients in MAST2 high expression group had worse OS (*p* < 0.0001; Fig. 3A). Subgroup analysis further indicated expression of MAST2 significantly decreased the OS of patients in stage G1/G2 (*p* < 0.0001), stage I/II (*p* = 0.036), stage III/IV (*p* = 0.0011), age of young (*p* = 0.00017) and old (*p* = 0.0038) and male (*p* < 0.0001). Since there is data on a large number of HCC samples, we performed a subgroup analysis among HCC tumors only and found the same results, which were also verified by GSE54236 and ICGC database (Fig. 3J–L).

Univariate analysis selected several variables correlated with OS, including stage (*p* = 0.001), T classification (*p* < 0.001), residual tumor (*p* = 0.003) and expression of MAST2 (*p* < 0.001). Together with T classification (*p* < 0.001) and residual tumor (*p* = 0.006), MAST2 expression (HR = 2.110, 95%CI: 1.467–3.035, *p* = 0.000) was independent risk factor for OS in liver cancer patients (Table 3) after adjusting the other variables correlated with OS (stage, T classification, and residual tumor).

**Table 3:** Univariate and multivariate analysis of overall survival.

Characteristics	Univariate analysis			Multivariate analysis		
	Hazard Ratio	95%CI (lower-upper)	*p*-value	Hazard Ratio	95%CI (lower-upper)	*p*-value
age (≥55/<55)	0.999	0.689–1.449	0.997			
gender (male/female)	0.801	0.562–1.142	0.220			
histological_type (hepatocholangiocarcinoma/hepatocellular/fibrolamellar)	0.989	0.267–3.665	0.986			
histologic_grade (G4/G3/G2/G1)	1.044	0.839–1.299	0.698			
clincial_stage (IV/III/II/I)	1.381	1.148–1.660	**0.001**	0.838	0.672–1.044	0.116
T_classification (T4/T3/T2/T1/NX)	1.662	1.387–1.990	**0.000**	1.844	1.459–2.331	**0.000**
N_classification (N1/N0/NX)	0.727	0.506–1.046	0.086			
M_ classification (M1/M0/MX)	0.716	0.495–1.037	0.077			
radiation_therapy (yes/no)	0.515	0.258–1.028	0.060			
residual_ tumor (RX/R2/R1/R0)	1.424	1.126–1.801	**0.003**	1.411	1.105–1.802	**0.006**
MAST2 (high/low)	2.248	1.572–3.215	**0.000**	2.110	1.467–3.035	**0.000**

Note: Bold values represent *p* < 0.05. CI, confidence interval. The table is partly similarity with previous publications in form^8–11^.

### Expression of MAST2 is associated with RFS

**Figure 4:**
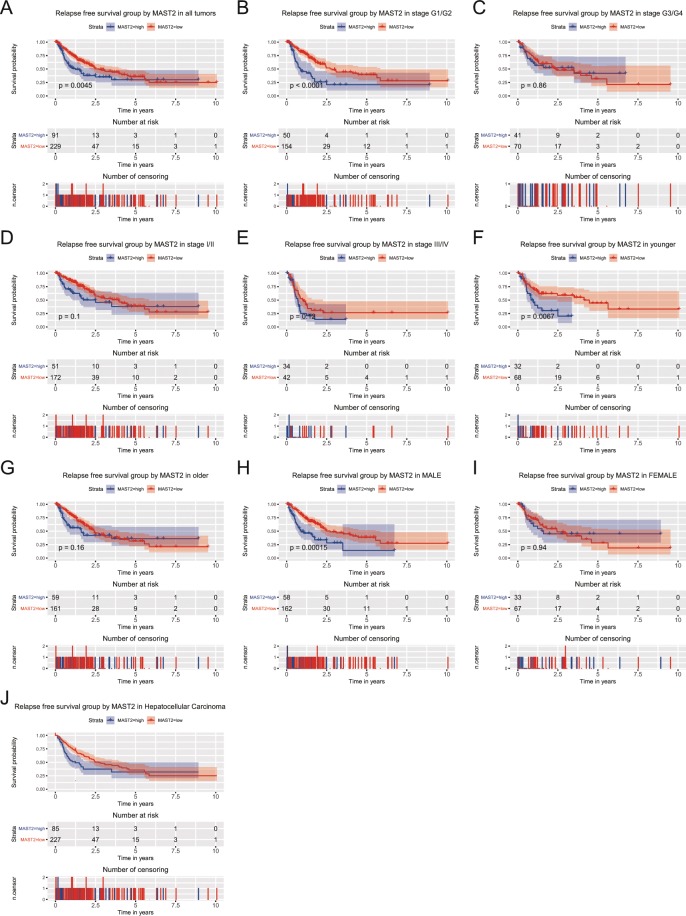
Kaplan-Meier curves for RFS in liver cancer. Kaplan-Meier curves for RFS in liver cancer for all patients, and patients in subgroup of stage G1/G2, stage G3/G4, stage I/II, stage III/IV, younger, older, male, female and HCC.

Kaplan-Meier curves indicated patients in group of high MAST2 expression exhibited worse RFS (p = 0.0045; Fig. 4). Moreover, patients in stage G1/G2 (*p* < 0.0001), younger (*p* = 0.0067) and male (*p* = 0.00015) were more sensitive to the poor prognostic effects of MAST2 high expression (Fig. 4). Subgroup analysis among HCC tumors only and found the same results (Fig. 4J). Univariate analysis selected that stage (*p* < 0.001), T classification (*p* < 0.001), residual tumor (*p* = 0.042) and expression of MAST2 (*p* = 0.005) were associated with RFS. In addition, multivariate analysis indicated MAST2 expression was an independent risk factor for RFS in liver cancer patients (HR = 1.517, 95%CI: 1.059–2.172, *p* = 0.023; Table 4).

**Table 4:** Univariate and multivariate analysis of relapse-free survival.

Characteristics	Univariate analysis			Multivariate analysis		
	Hazard Ratio	95%CI (lower-upper)	*p*-value	Hazard Ratio	95%CI (lower-upper)	*p*-value
age (≥55/<55)	0.898	0.631–1.278	0.550			
gender (male/female)	0.992	0.696–1.415	0.966			
histological_type (hepatocholangiocarcinoma/hepatocellular/fibrolamellar)	2.024	0.656–6.24	0.220			
histologic_grade (G4/G3/G2/G1)	0.985	0.801–1.21	0.883			
clincial_stage (IV/III/II/I)	1.656	1.379–1.988	**0.000**	1.114	0.862–1.439	0.410
T_classification (T4/T3/T2/T1/NX)	1.778	1.494–2.117	**0.000**	1.635	1.255–2.13	**0.000**
N_classification (N1/N0/NX)	0.971	0.674–1.399	0.874			
M_ classification (M1/M0/MX)	1.172	0.789–1.742	0.432			
radiation_therapy (yes/no)	0.742	0.256–2.156	0.584			
residual_ tumor (RX/R2/R1/R0)	1.275	1.009–1.612	**0.042**	1.335	1.054–1.692	**0.017**
MAST2 (high/low)	1.663	1.166–2.372	**0.005**	1.517	1.059–2.172	**0.023**

Note: Bold values represent *p* < 0.05. CI, confidence interval. The table is partly similarity with previous publications in form^8–11^.

## Discussion

Liver cancer malignant tumor with poor prognosis, which is predominantly due to the fact that diagnosis is often made late or inaccurate^2^. To identify a new biomarker for early and accurate diagnosis has great clinical significance, many researchers have been working on developing novel biomarkers in liver cancer^8–11^. In this study, we explored the diagnostic and prognostic role of MAST2 in liver cancer patients. We found that MAST2 highly expressed in liver cancer and thus, may have diagnostic value for this cancer, and its expression was correlated with histological type, histologic grade, stage, T classification, N classification, and survival status. Moreover, high MAST2 expression was associated with poor OS and RFS in patients, which suggested the prognostic role of MAST2 in liver cancer.

MAST2, as a microtubule associated kinase, plays important roles in a wide range of life activities. Previous studies have reported the role of MAST2 in evolution^12^, marfan syndrome^13^, neurodegeneration^14^, rabies virus infection^15^, nonobstructive azoospermia^16^, experimental autoimmune encephalomyelitis^17^, chronic myeloid leukemia^18^ and breast cancer^4^. Our studies showed the abnormal expression and prognostic effects of MAST2 in liver cancer, which broadened the field of scientific research on MAST2.

The upregulation of MAST2 has been reported in several tumors, including esophageal cancer, pancreatic cancer, sarcomas^5^, chronic myeloid leukemia^18^ and breast cancer^4^. Our results showed the overexpression of MAST2 in liver cancer. It is consistent with previous reports. We also found that the upregulation of MAST2 was distinct in different clinical features of liver cancer, such as histologic grade, stage and T classification. Moreover, the AUC of MAST2 suggest a potentially important value in tumor diagnosis and prognosis.

The effect of MAST2 in promoting tumor cell proliferation has been reported in glioblastoma. Eissmann *et al*. used lentiviral shRNA transduction in U87 cell line not only resulted in significantly increased apoptosis and decreased cell proliferation, but also delayed tumor growth^5^. The tumor promoting effects of MAST2 may provide a reasonable explanation for the phenomenon in our research that patients with advanced stage and worse status showed high MAST2 expression.

MAST2 plays its role through binding the C-terminal of PTEN with its PDZ domain. PTEN regulates multiple cellular processes, including polarity, migration, proliferation and metabolism^19^. PTEN, also as a tumor suppressor gene, its aberrant expression is associated with tumorigenesis and progression^20^. In our study, the poor prognosis of patients with high MAST2 expression might due to the aberrant function of PTEN.

This study firstly demonstrates the potentially diagnostic and prognostic significance of MAST2 in liver cancer patients. Moreover, the distinct expression of MAST2 and prognosis in subgroups by clinical features also provided multiple guidelines of precision therapy. However, the lower expression and AUC of MAST2 in stage IV might result from the limited sample size of stage IV patients, further studies are needed to verify these findings.

In conclusion, our study found upregulation of MAST2 in liver cancer, which corresponded with tumor progression and poor prognosis. Our findings suggest MAST2 could be a novel diagnostic and prognostic biomarker for liver cancer patients.

## Material and Methods

### Data mining

The characteristics and gene expression in patients with liver cancer were downloaded from TCGA database (https://cancergenome.nih.gov/), GEO database (https://www.ncbi.nlm.nih.gov/gds/) and ICGC database (https://icgc.org/). All data were analyzed by R (version 3.5.3)^21^.

### Statistical analysis

Boxplots were used to illustrate the gene expression differences between different groups and subgroups through ggplot2^22^. ROC curve was applied to examine the diagnostic capability of MAST2 in liver cancer^23^. Chi-square and Fisher test were used to explore the association between patients’ characteristics and MAST2 expression. Survival curves were applied to explore OS and RFS of patients in different MAST2 expression group through Survival package^24^. Univariate analysis was used to select variables relating to outcomes. Multivariate analysis was applied to investigate the influence of MAST2 expression on OS and RFS of patients with liver cancer. The methodological is partly similarity with previous publications^8–11^.

## Data Availability

All data generated or analyzed during this study are included in this published article.
